# Supplementary material for: The relative contribution of color and material in object selection
Source: PLoS Comput Biol. 2019 Apr 12;15(4):e1006950. doi: 10.1371/journal.pcbi.1006950 (PMC6490924; doi:10.1371/journal.pcbi.1006950)
Supplement: S1 Appendix — (DOCX) [file pcbi.1006950.s001.docx]

# S1 Appendix. Deviations from and additions to preregistered plan for data collection and analysis

Below we summarize deviations from the preregistered plan for data collection and analysis and we list all additional exploratory analyses we reported here that were not included in the preregistered plan.

Changes in experimental procedures:

- One observer was recruited but excluded from the experiment before completion due to non-compliance with experimental instructions (see Methods). Data from this observer were not analyzed.

Additional (exploratory) data analyses, not included in the preregistered data analysis plan:

- We plot color-material trade-off functions and data from the subset of trials in which the tests were a material match and a color match to describe the quality of the model fit to the data. Color-material trade-off functions and the corresponding data were used in this way for the preliminary experiment [21].
- We compared the quality of the fit of the two best models based on two distance metrics by conducting a 2-tailed paired t-test on cross-validated log-likelihoods. We also quantified the general agreement between the color-material weights inferred from the best models based on different distance metrics by computing a Spearman rank-order correlation coefficient.
- To investigate the relationship between the parameters describing the stimulus representation and the color-material weight, we used the bootstrapped data to compute color-material slope ratios and compare them to recovered color-material weights. We illustrate this relationship both graphically (Figure 6) and quantitatively (Pearson correlation coefficients).
