# Supplementary material for: The relative contribution of color and material in object selection
Source: PLoS Comput Biol. 2019 Apr 12;15(4):e1006950. doi: 10.1371/journal.pcbi.1006950 (PMC6490924; doi:10.1371/journal.pcbi.1006950)
Supplement: S2 Appendix — (DOCX) [file pcbi.1006950.s002.docx]

**S2 Appendix. Experimental instructions.**

Below we provide experimental instructions verbatim. These were read to the observer at the beginning of the first experimental session. At the beginning of each following session the observers were only reminded of the task (experimenter only read the paragraph that begins with “Your task…”).

“During the experiment we will ask you to view the stimuli from the position indicated by this chin rest. At this time, please adjust the chair so that your chin is positioned at the chin rest, your forehead is leaning against the upper bar and you are sitting comfortably with your back straight. We will describe your task in the experiment using a couple of training trials.”

The experimenter starts the training, which consists of four trials (across trials the test that is identical to the target was paired with competitors C_-3_M_0_, C_+3_M_0_, C_0_M_-3_ and C_0_M_+3_). Training data is not analyzed.

“On each trial three scenes will be presented on the screen, each containing one object.

The object in the middle scene is the target for the trial. The objects in the left and in the right scene are the test objects.”

“Your task is to select the test object that is most similar to the target. On some trials, both tests will be very similar to the target. Always try to pick the one that is most similar.”

“You will use this joystick to provide the response. To select the test on the left, press button 1 on the left. To select the test on the right, press button 3 on the right. When you make the choice a small black dot will briefly flash just above the test object you selected. At this point the trial will end and the new set of objects will appear on the screen.”

“There are 270 trials within a block and they are divided into 9 sets of trials. After each set, you will have a chance to take a break if you need one. The experiment will pause and a voice will inform you how many sets in this block are done and how many are left. You can take as much time as you need to rest between the sets. When you want to continue, press one of the front buttons on the joystick and the next trial will start. After a block of trials is finished, the screen will turn gray. At that point, we will take a 5-10-minute break before we continue with the second block of trials. Today we will do 2 blocks of trials.”

“It is very important that your head is positioned at the chin rest while you are viewing the stimuli. Please make sure you remain in this position during the set of trials. You will be able to move, stretch or adjust your position in between the sets or between the blocks. Do you have any questions?

“I will now initiate the experiment and leave the room. The experiment will start shortly after.”

Experimental program pauses for 15 seconds before the first trial to allow experimenter to leave the room.
